# Influence of the Spatial Dimensions of Ultrasonic Transducers on the Frequency Spectrum of Guided Waves

**DOI:** 10.3390/s17081825

**Published:** 2017-08-08

**Authors:** Vykintas Samaitis, Liudas Mažeika

**Affiliations:** Prof. K.Baršauskas Ultrasound Research Institute, Kaunas University of Technology, K. Baršausko St. 59, LT-51423 Kaunas, Lithuania; ui@ktu.lt

**Keywords:** structural health monitoring, ultrasonic guided wave excitation, excitability function, frequency response, MFC transducer

## Abstract

Ultrasonic guided wave (UGW)-based condition monitoring has shown great promise in detecting, localizing, and characterizing damage in complex systems. However, the application of guided waves for damage detection is challenging due to the existence of multiple modes and dispersion. This results in distorted wave packets with limited resolution and the interference of multiple reflected modes. To develop reliable inspection systems, either the transducers have to be optimized to generate a desired single mode of guided waves with known dispersive properties, or the frequency responses of all modes present in the structure must be known to predict wave interaction. Currently, there is a lack of methods to predict the response spectrum of guided wave modes, especially in cases when multiple modes are being excited simultaneously. Such methods are of vital importance for further understanding wave propagation within the structures as well as wave-damage interaction. In this study, a novel method to predict the response spectrum of guided wave modes was proposed based on Fourier analysis of the particle velocity distribution on the excitation area. The method proposed in this study estimates an excitability function based on the spatial dimensions of the transducer, type of vibration, and dispersive properties of the medium. As a result, the response amplitude as a function of frequency for each guided wave mode present in the structure can be separately obtained. The method was validated with numerical simulations on the aluminum and glass fiber composite samples. The key findings showed that it can be applied to estimate the response spectrum of a guided wave mode on any type of material (either isotropic structures, or multi layered anisotropic composites) and under any type of excitation if the phase velocity dispersion curve and the particle velocity distribution of the wave source was known initially. Thus, the proposed method may be a beneficial tool to explain and predict the response spectrum of guided waves throughout the development of any structural health monitoring system.

## 1. Introduction

Ultrasonic guided waves (GWs) have shown great potential in the structural health monitoring (SHM) of various ageing engineering components to ensure their safe, reliable, and optimal performance [1,2]. GWs are sensitive to changes in the elastic modulus of the material and possess minor amplitude damping, which enables the inspection of large structures using only a few transducers and the detection of both surface and internal defects in almost real-time [3]. Lamb waves are a kind of guided wave that propagate in plate-like structures in cases where the plate thickness is considerably lower than the wavelength. Lamb waves provide high interrogation volumes and are sensitive to defects of different kinds, hence many studies have been conducted on the application of Lamb waves to detect delaminations [4,5,6,7,8], cracks [9,10,11], notches [12,13,14], impact damages [15,16,17,18,19], and other structural non-homogeneities. The application of Lamb waves in such a wide variety of areas, all looking for different kinds of defects, shows the huge potential of GW inspection. However, as Lamb waves possess a multi-modal, dispersive, and mode converting nature, complicated signal analysis is a key issue limiting the development of reliable inspection systems. The signals captured from the structure are often distorted and overlapped, while the damage-scattered components are concealed within. To simplify guided wave inspection, various approaches have been used to generate Lamb waves with the desired dispersive properties, sensitivity to defects, excitability, and detectability [20]. Of these, piezoelectric wafers [21,22,23], interdigital transducers [24,25,26,27,28,29] and phased-delay excitation [30,31,32] have attracted the most attention.

A proper understanding of the response of various ultrasonic transducers has been of great interest among various researchers. Giurgiutiu et al. [33,34,35] completed extensive work on analyzing the behavior of piezoelectric wafer active sensors (PWAS) where they used a 2D plane strain model based on the integral transform method and demonstrated that maximal response amplitudes of PWAS were at frequencies, where the width of transducer was equal to odd multiple half wavelengths. In the same fashion, for the even number of half wavelengths response minima can be found. Due to different velocities, the response of A_0_ and S_0_ modes of Lamb waves were different, hence the desired mode could either be enhanced or suppressed by selecting the appropriate size of the PWAS. Similarly, Raghavan and Cesnik [36] analyzed the displacement response of a piezo-disc actuator as a function of its length. Grondel et al. [37,38] used a normal mode expansion method to investigate selective A_0_ mode excitation using surface bonded piezoceramic transducers. Comprehensive research on Lamb wave excitation was presented by Lanza di Scalea et al. [39] where they analyzed the response of surface bonded transducer to Rayleigh and Lamb waves by employing both harmonic and pulse excitation. Zeng et al. [40] analyzed waveform distortions caused by the amplitude dispersion of guided waves and established a method based on the Vold–Kalman filter and Taylor series expansion to remove such effects. Finally, Schubert et al. [41] focused on the impact of amplitude dispersion and sensor size on the response of PWAS attached to composite structures where the authors combined analytical excitability functions, phase velocity, attenuation curves and geometric influences of the transducer to predict the output signal of PWAS.

Some research related to the response of piezocomposite transducers, like macro-fiber composites (MFC) [42] can also be found in the literature. MFCs are directional transducers made of uniaxially aligned rectangular cross-section fibers, surrounded by a polymer matrix and comb type electrode pattern [20]. They provide good surface conformability due to the piezocomposite structure, are wideband, non-brittle, thin and low-cost, which makes them a perfect choice for structural health monitoring applications. Matt et al. [43] proposed an approach of MFC rosettes for passive damage localization, which allowed them to extract the direction of the incoming wave. Throughout the development of the rosette approach, the authors estimated the frequency response of a MFC transducer at different wave propagation angles. Furthermore, the authors demonstrated that the frequency response of Lamb waves excited by a MFC transducer showed local minima and maxima, which was related to the length of the transducer and properties of the medium. Birchmeier et al. [44] analyzed the generation of Lamb waves using active fiber composite (AFC) transducers and defined transfer functions for the A_0_ and S_0_ modes of Lamb waves and related its oscillations with the length of the element and properties of the wave in the medium. 

The research mentioned above has demonstrated that the amplitude of guided wave modes is a function of frequency and transducer dimensions, which means that the frequency response is different for every guided wave mode due to different transducer size to wavelength ratio. As the wavelength varies from one material to another, the response also becomes material-dependent. In such cases, the result of wave interference and interaction with damage will vary depending on source type, size, and dispersive properties of the medium. In cases where multiple modes are present in the structure, the signal analysis and interpretation becomes even more complicated. To develop reliable structural health monitoring systems, the source influence on the response spectrum of guided wave modes must always be addressed. The aim of this study was to propose and verify an analytical method to help predict the response spectrum of guided wave modes based on initial knowledge on transducer type, size, and propagation medium. First, the scientific problem is explained using the general theory of ultrasonic guided waves, and the proposed excitability function estimation technique is formulated based on the Fourier analysis of particle velocity distribution on the excitation area. Then, numerical simulations and different experiments are conducted to validate and investigate the feasibility of the proposed technique. Strong experimental evidence is provided about the influence of transducer vibration and size to the frequency spectrum of guided waves.

## 2. Statement of the Problem 

One of the most important and versatile features used to describe the modes of guided waves is frequency response. The frequency response of guided wave modes depends on both the structural properties of the investigated medium and the parameters of the source, such as size and type of excitation. In most theoretical models, it is presumed that the conditions ideally possess the uniform loading and continuous plane wave excitation. However, in reality, the transducers possess limited size and frequency bandwidth. Hence, the type of excitation determines the mode which is introduced into the structure, whereas the amplitude of vibrations depends on the size-wavelength ratio of the source.

**Figure 1:**
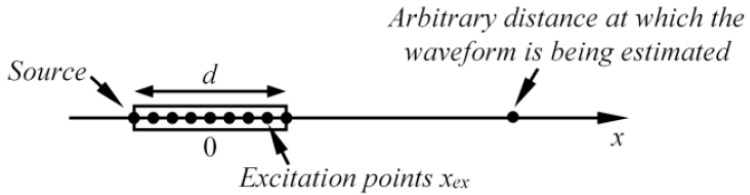
The analyzed 1D guided wave excitation case.

The generation mechanism of GW is different compared to the bulk wave case. As both bulk and guided waves may be generated employing the same principle (i.e., angle beam excitation), whether a bulk or guided wave is generated mainly depends on the frequency to sample thickness ratio. To excite GWs, the wavelength must be greater than the thickness of the material, while bulk waves generally propagate in the infinite medium where the boundaries only have a minor influence on wave propagation. Due to this reason, in the case of bulk waves, there are mostly one or two desired non-dispersive waves propagating at constant phase velocity. In contrast, the GW is a superposition of longitudinal and shear waves, which reflect back and forth and convert to other modes. At a given frequency, the results of such wave interaction may be either constructive, destructive, or intermediate. As a consequence, different GW modes are produced simultaneously, each having its own frequency-thickness dependent propagation velocity and unique distribution of particle velocity across the thickness of the sample. The response of the ultrasonic transducer (due to diffraction) also depends on the direction of wave generation. It is common to calculate the response function along the transducers axis; however, waves can also be generated in other directions. For the sake of better understanding, GW excitation in a 1D approach was analyzed, and is presented in Figure 1.

In this case, the non-dispersive mode is excited using a point type transducer and propagates along the plate with velocity c. According to general theory of ultrasonic guided waves and the governing wave equations, the waveform at arbitrary distance along the x axis can be expressed as [45,46]:

(1) $$u\left(t,x\right)=u_{ex}\left(t-x/c\right),$$

where u_ex_(t) is the waveform of particle velocity generated at excitation point (x = 0). If the transducer possesses finite dimensions, then Equation (1) can be re-written as:

(2) $$u\left(t,x\right)=\int_{-d/2}^{d/2}u_{ex}\left(t-\left(x-x_{ex}\right)/c,x_{ex}\right)dx_{ex},$$

where d is the length of transducer; x_ex_ is the position of excitation point on x axis. Assume that $u_{ex}\left(t,x_{ex}\right)=A\left(x_{ex}\right)\cdot u_{ex}\left(t-x_{ex}/c\right)$, where A(x_ex_) is the amplitude of the waveform at the excitation point x_ex_. From this assumption, it follows that the excitation waveform is the same at all excitation points; however, it possesses different amplitudes at each node. Next, Equation (2) can be reorganized as follows:

(3) $$u\left(t,x\right)=\int_{-d/2}^{d/2}A\left(x_{ex}\right)\cdot u_{ex}\left(t-\left(x-x_{ex}\right)/c\right)dx_{ex}.$$

Assuming that x_ex_ = τ·c, Equation (3) can be further reorganized as:

(4) $$u\left(t,x\right)=c\cdot \int_{-d/2c}^{d/2c}A\left(c\tau \right)\cdot u_{ex}\left(\left(t-x/c\right)-\tau \right)d\left(\tau \right).$$

Equation (4) is the convolution integral, which can be solved using Fourier transform:

(5) $$u\left(t,x\right)=FT^{-1}\left\{FT\left[A\left(c\tau \right)\right]\cdot FT\left[u_{ex}\left(\left(t-x/c\right)\right)\right]\right\},$$

where FT and FT^−1^ denotes Fourier and inverse Fourier transform respectively. Term FT[A(cτ)] can be called the spatial filter or excitability function as it is only related to the distribution of the excitation amplitudes. This excitability function was investigated in this study. In the case dispersive waves are excited using point type transducer, the waveform at arbitrary distance x can be expressed as convolution of input signal and system’s impulse response [45,47]:

(6) $$u\left(t,x\right)=FT^{-1}\left\{FT\left[u_{ex}\left(t\right)\right]\cdot e^{-\frac{j\alpha \omega x}{c_{p}\left(\omega \right)}}\right\},$$

where α is attenuation; ω = 2πf; f is the frequency; and c_p_(ω) is the frequency dependent phased velocity dispersion curve of the mode under analysis. In the case where a transducer possesses finite dimension and the excitation of the corresponding component of particle velocity is not uniform, each point has different waveform u_ex_(t,x_ex_):

(7) $$u\left(t,x\right)=\int_{-d/2}^{d/2}FT^{-1}\left\{FT\left[u_{ex}\left(t,x_{ex}\right)\right]\cdot e^{-\frac{j\alpha \omega \left(x-x_{ex}\right)}{c_{p}\left(\omega \right)}}\right\}dx_{ex}.$$

If it is assumed that the excitation waveforms in the transmitter area is the same and from point to point differs only in amplitude, it means $u_{ex}\left(t,x_{ex}\right)=A\left(x_{ex}\right)\cdot u_{ex}\left(t-x_{ex}/c\right):$

(8) $$u\left(t,x\right)=\int_{-d/2}^{d/2}FT^{-1}\left\{FT\left[A\left(x_{ex}\right)\cdot u_{ex}\left(t\right)\right]\cdot e^{-\frac{j\alpha \omega \left(x-x_{ex}\right)}{c_{p}\left(\omega \right)}}\right\}dx_{ex},$$

then:

(9) $$u\left(t,x\right)=\int_{-d/2}^{d/2}A\left(x_{ex}\right)\cdot FT^{-1}\left\{FT\left[u_{ex}\left(t\right)\right]\cdot e^{-\frac{j\alpha \omega \left(x-x_{ex}\right)}{c_{p}\left(\omega \right)}}\right\}dx_{ex}.$$

It follows from Equation (9) that the waveform of generated waves and their frequency response depends not only on the frequency spectrum of the excitation signal (in most cases assumed as the transducer characteristic), but also on excitation force distribution along the wave propagation direction. If the conventional thickness mode transducer is used to generate the guided waves, which propagates along the sample, the frequency response of the generated waves will be different from conventional frequency response of transducer determined using bulk waves. Additionally, it will be different for each mode of the guided waves. Thus, the aim of this study was to develop analytical method to help predict the response spectrum of guided wave modes.

## 3. Theoretical Model for the Prediction of Frequency Spectrum of Generated Guided Waves

**Figure 2:**
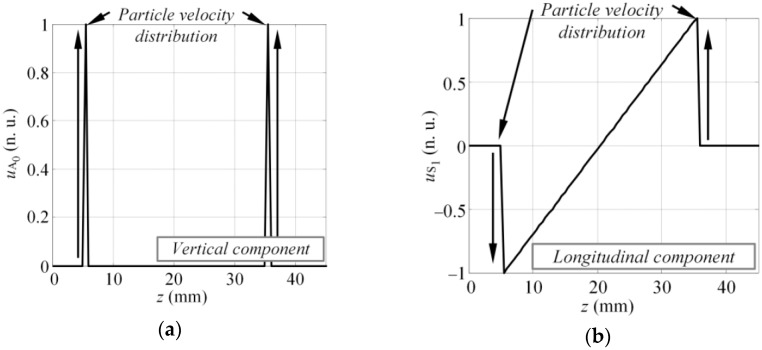
The presumed spatial distribution of the particle velocity of the MFC transducer on the excitation surface: the vertical or normal component, which mainly generates the A_0_ mode (**a**) and the longitudinal or tangential component, which produces the S_1_ mode (**b**).

The method proposed in this study is based on Fourier analysis of the particle velocity distribution (u) on the excitation surface at the initial instant of excitation. Such particle velocity distribution is related to the excitation force, which is required to introduce the desired mode into the structure. Assuming that the virtual MFC transducer is mounted on the surface of the sample and excites the A_0_ and S_1_ modes simultaneously, at the initial instant of excitation, the spatial distribution of the particle velocity on the excitation area can be described as Figure 2. The spatial distribution of particle velocity presented in Figure 2 are in 2D and it is presumed to be uniform across the width of the transducer. The spatial distribution of particle velocity was deliberately selected based on the vibration of MFC transducer operating at elongation d_33_ mode. Hence, it is presumed that the out-of-plane component of the particle velocity at the excitation surface had to be exclusively concentrated at the edges of the transducer to introduce the A_0_ mode (Figure 2a). Similarly, the saw tooth like in-plane component of the particle velocity distribution caused by elongation of the MFC transducer generated the S_1_ mode (Figure 2b). Mathematically the distribution presented in Figure 2 can be expressed as follows:

(10) $$u_{A_{0}}\left(z\right)=\{\begin{array}{cc} 1 & z=z_{1}\;or\;z=z_{2}\\ 0 & other\;case \end{array},$$

(11) $$u_{S_{1}}\left(z\right)=\{\begin{array}{cc} \frac{2z-l}{l} & z_{1}<z<z_{2}\\ 0 & other\;case \end{array},$$

where z_1_ and z_2_ are the coordinates of the front and back edge of the MFC transducer; and l = z_2_ − z_1_ is the length of transducer. Equations (10) and (11) describe the spatial distribution of particle velocity, which is graphically illustrated in Figure 2a,b, respectively. Such distribution was selected based on a-priori knowledge of the vibration of a MFC (M-2814 P1) type transducer.

For non-dispersive waves, spatial particle velocity distribution can be transformed to the particle velocity time domain as u(t), using the simple relation t = z/c_p_, where c_p_ is the phase velocity. For dispersive waves, phase velocity is the function of frequency c_p_(f), hence this transformation becomes frequency dependent u(z/c_p_(f)). The excitability function or the response amplitude at the given discrete frequency f_k_ can be expressed as the magnitude of the Fourier representation of particle velocity distribution u(z/c_p_(f_k_)). The same procedure must be repeated over the bandwidth of the transducer to collect the whole set of magnitude values of excitability function:

(12) $$H_{A_{0}}\left(f_{k}\right)={U_{A_{0}}^{\prime }\left(f\right)|}_{f=f_{k}},\;U_{A_{0}}^{\prime }\left(f\right)=\left|FT\left[u_{A_{0}}\left(\frac{z}{c_{pA_{0}}\left(f_{k}\right)}\right)\right]\right|,$$

(13) $$H_{S_{1}}\left(f_{k}\right)={U_{S_{1}}^{\prime }\left(f\right)|}_{f=f_{k}},\;U_{S_{1}}^{\prime }\left(f\right)=\left|FT\left[u_{S_{1}}\left(\frac{z}{c_{pS_{1}}\left(f_{k}\right)}\right)\right]\right|,$$

where $H_{A_{0}}$(f_k_) and $H_{S_{1}}$(f_k_) are the analytical excitability functions for the A_0_ and S_1_ modes; $u_{A_{0}}$ and $u_{S_{1}}$ are the particle velocity distributions for A_0_ and S_1_ modes at particular frequency f_k_; and FT denotes the Fourier transform. The above-mentioned excitability function is the transfer function of the ultrasonic transducer, which defines how efficiently it will generate the particular mode at a given frequency and transducer size. The method proposed in this study was not limited to any particular transducer or material and can be used to predict the excitability function on any structure, under any type of excitation. To obtain proper results, the method required the phase velocity dispersion curve of the analyzed structure and the operation principle of the investigated transducer (particle velocity distribution for the analyzed mode) as input data.

**Figure 3:**
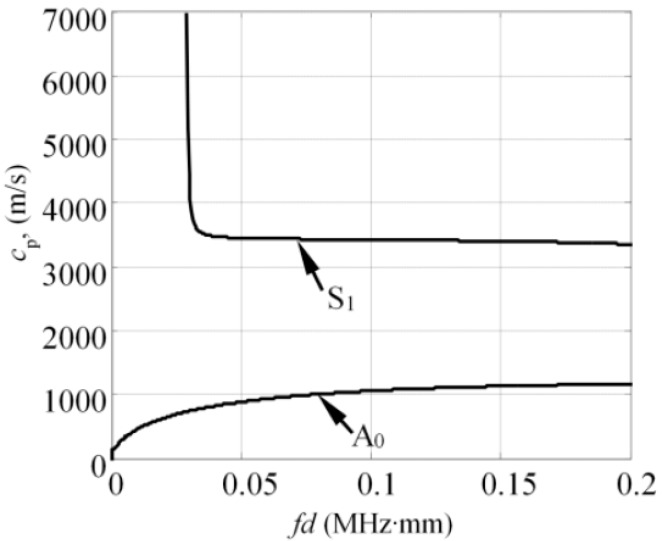
The phase velocity dispersion curves of 4 mm thick and 70 mm wide glass fiber reinforced plastic (GFRP) plate, calculated using the semi analytical finite element method.

**Figure 4:**
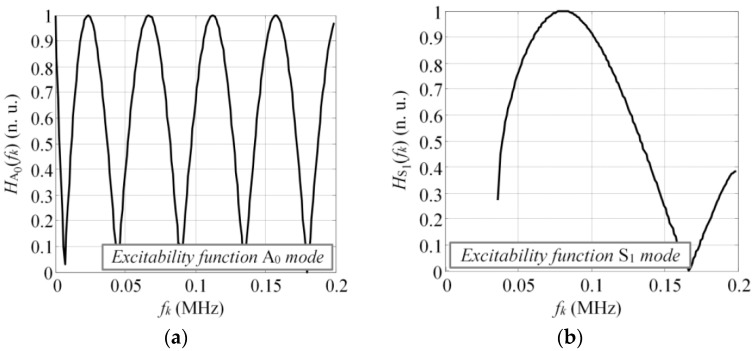
The excitability functions on 4 mm thick GFRP plate for the A_0_ (**a**) and S_1_ (**b**) modes, calculated according to the spatial distribution of the vertical and longitudinal component of particle velocity.

To estimate the excitability function as per Equations (12) and (13), consider a 4 mm thick and 70 mm wide glass fiber reinforced plastic (GFRP) plate as an investigated sample, with the material properties as follows: Young’s modulus (E_x_ = 10 GPa, E_z_ = 35.7 GPa); Poisson’s ratio (υ_xz_ = 0.325, υ_zx_ = 0.091, υ_yx_ = 0.35); Shear modulus (G_xz_ = 2.8 GPa; density: ρ = 1800 kg/m^3^). The dispersion curves (DC) of the analyzed structure can be seen in Figure 3 where the dispersion curves presented were validated experimentally and corresponded to the properties of the real mock-up sample, which was used for the experiments in this study. The analytically obtained excitability functions for the A_0_ and S_1_ modes can be seen in Figure 4a,b. The excitability functions presented below were estimated considering the same particle velocity distribution as defined by Equations (10) and (11).

The results presented in Figure 4 indicated that in both cases the response amplitude of the excitability function oscillated with an increase of frequency. The zero values of the excitability function were found at some frequency components. The periodicity of response amplitude oscillation was higher for the A_0_ mode, meaning that the modes possessing short wavelengths were likely be more distorted in comparison to the symmetrical modes. These excitability curves can be used to enhance or suppress the excitation of the desired mode; however, as the relative broadband excitation is usually used to drive the transducer, multiple modes are being generated anyway. In such cases, depending on the frequency and the bandwidth of the excitation pulse, the waveforms of at least asymmetrical modes will be distorted due to the impact of the excitability function. Thus, the influence of the excitability function must be considered while developing methods for SHM.

**Figure 5:**
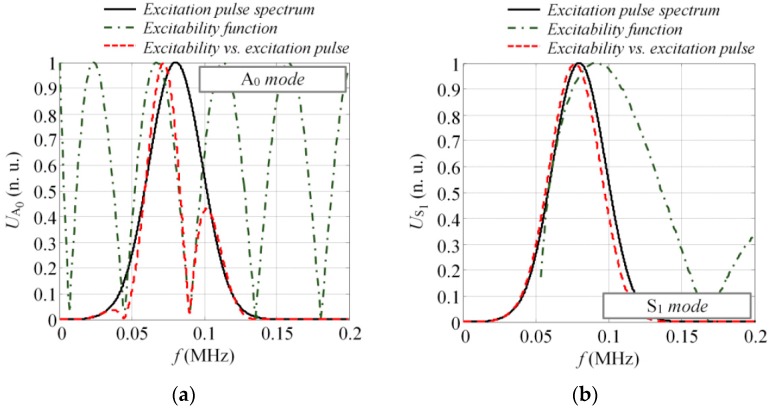
The influence of the excitability function to the frequency response of the guided waves for the asymmetric A_0_ (**a**) and symmetric S_1_ (**b**) modes (solid line: spectrum of the excitation pulse, dash-dot line: excitability function, dashed line: product of excitation pulse and excitability function).

To illustrate the influence of excitability function on the frequency spectrum of guided wave modes, we assumed that the transducer was driven by a tone burst with a Gaussian envelope of three periods and central frequency of 80 kHz. The length of the transducer was 28 mm along the wave propagation direction and had a particle velocity distribution as shown in Figure 2. Next, the magnitude spectrum of the excitation pulse looked like that represented by the solid line in Figure 5, which also shows the excitability function and its product with the magnitude spectrum of excitation pulse as dash-dot and dashed lines, respectively. It was observed that under such excitation and size of the source, some significant filtering was present in the spectrum of the A_0_ mode (Figure 5a). Meanwhile, the S_1_ mode was not filtered due to the significantly larger wavelength compared to the A_0_ mode (45.5 mm versus 13.1 mm @ 80 kHz). To correctly interpret the guided wave propagation, mode interference, and interaction with defects, the likely filtering effects must be considered, although it’s usually still neglected in most research.

## 4. Validation of the Proposed Method

The goal of this section was to validate the excitability function estimation technique presented previously and to verify that the magnitude spectrum of each guided wave mode present in the structure can be expressed as a product of the frequency spectrum of the excitation pulse and the excitability function. For this purpose, the 3D finite element (FE) model of the transient wave propagation and the experimental investigation was performed on the GFRP sample.

### 4.1. Validation of the Proposed Method Employing Finite Element (FE) Model

The 3D finite element (FE) model of the transient wave propagation was employed for a complex shaped GFRP plate with dimensions of 800 mm × 70 mm × 4 mm. Next, the frequency spectrum of the fundamental A_0_ and S_1_ modes were extracted from the FE simulation data and compared to the analytically predicted excitability functions. In the upcoming chapters, the numerical model of the GFRP sample will be briefly described, followed by the procedure used to extract the spectrum of A_0_ and S_1_ modes. The chapter will be concluded with the comparison between the spectra of A_0_, S_1_ modes and analytical excitability functions.

#### 4.1.1. Description of the Glass Fiber Reinforced Plastic (GFRP) FE Model

**Figure 6:**
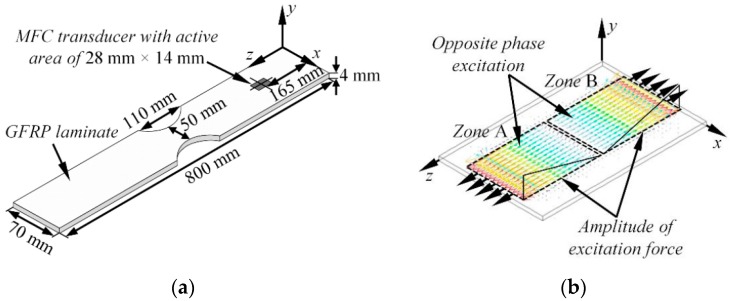
The schematic view of the GFRP plate used in FE simulations (**a**), the principle of guided wave generation in the FE model simulating the d_33_ mode of MFC transducer (**b**).

The graphic representation of the model used in FE simulations can be seen in Figure 6a. The geometrical shape and dimensions of the simulated sample were deliberately selected to correspond to the real mock-up specimen, which was used later for experimental verification. In the numerical model, the operation of the MFC (M-2814 P1) actuator, with an active area of 28 mm × 14 mm working in the elongation d_33_ mode was simulated. The distance along the z axis between the center of the virtual transducer and the nearest end of the sample was set to 165 mm. The excitation area was aligned to the center of the sample with respect to the x axis. The thin layer of wax (Young’s modulus: E = 1.81 GPa, Poisson’s ratio: υ = 0.49; density: ρ = 951 kg/m^3^) was used as a coupler between the specimen and the transducer. To simulate the operation of the MFC transducer in the elongation d_33_ mode, the active surface of the actuator was divided into two separate areas referred to as Zone A and Zone B, each with dimensions of 14 mm × 14 mm (see Figure 6b). Then the excitation of guided waves was simulated by applying the monotonically increasing excitation force of the opposite phase to the nodal points in “zone A” and “zone B” as illustrated in Figure 6b. In this way, the waves generated in Zone A propagated along the positive direction of the z axis while the waves introduced in Zone B propagated backwards. Under this type of excitation, typically both fundamental asymmetrical and symmetrical modes are generated simultaneously. The waveform of the excitation force was a burst with a Gaussian envelop of three periods, a central frequency of 80 kHz and a bandwidth of 42.9 kHz at −6 dB.

**Figure 7:**
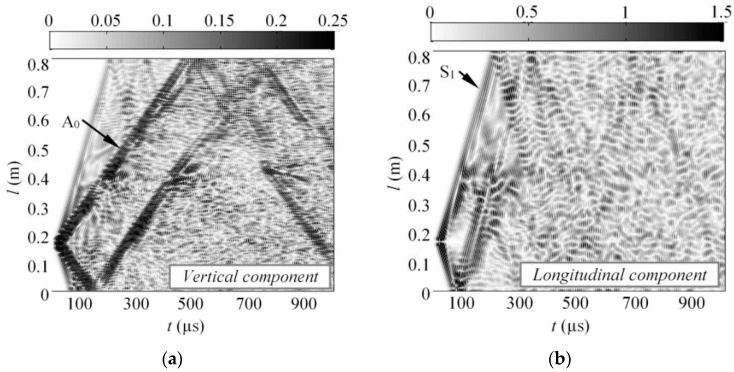
The simulated B-scan images of the vertical (**a**) and longitudinal (**b**) component of guided waves along the centerline of the GFRP sample.

For transient simulation, the Newmark time integration scheme was applied. The integration step in time domain was 0.625 µs, which is 1/20 of the period at 80 kHz central frequency. The sample was meshed using SOLID64 elements, which had eight nodes each with three degrees of freedom. The average spatial size of the element was equal to 1 mm possessing 14 nodes per wavelength for the slowest A_0_ mode at 80 kHz frequency. The finite elements of the regular areas were created using the mapped mesh, whereas for the regions close to cut-outs, the free sweep mesh was implemented. Material properties for the GFRP were selected as the same to those presented in the previous section. The variables monitored in this study was a vertical (y) and longitudinal (z) component of particle velocity at the nodal points situated along the centerline of the sample. The B-scan image of the vertical and longitudinal component of particle velocity can be seen in Figure 7.

At the end of the simulation routine, the frequency spectrum of the fundamental A_0_ and S_1_ modes present in the structure was extracted from the B-scan data of the vertical and longitudinal component of particle velocity. For this purpose, the B-scan data u(t,x) was transferred to the frequency-wavenumber domain u(f,ξ) using the 2DFFT approach [48]. The frequency-wavenumber representation of the B-scan showed the wavenumber DC for all modes available in the structure. As the fundamental A_0_ and S_1_ waves were the modes of interest, the appropriate DC were filtered from u(f,ξ) by employing the Gaussian filter, which mathematically can be described by the following equation:

(14) $$F\left(f,\xi \right)=e^{-A{\left(\xi -\xi_{0}\left(f\right)\right)}^{2}},$$

where A is the coefficient that determines the width of the Gaussian filter along wavenumber axis; $\xi_{0}\left(f\right)=a_{k}\cdot f+b_{k}$; a_k_ and b_k_ are the coefficients of the linear equation; k = 1 …K; K is the total number of linear points; f ∈ [f_k_,f_k+1_]; and f_k_, f_k+1_ defines the frequency bandwidth under investigation. The Gaussian filter was adjusted to fit the DC of each mode of interest, meaning that each mode was approximated by a set of linear segments in the frequency-wavenumber domain which were selected manually. As the consequence, the filtered A_0_ and S_1_ modes can be described by the equations:

(15) $$U_{A_{0}}\left(f,\xi \right)=u\left(f,\xi \right)\cdot F_{A_{0}}\left(f,\xi \right),$$

(16) $$U_{S_{1}}\left(f,\xi \right)=u\left(f,\xi \right)\cdot F_{S_{1}}\left(f,\xi \right),$$

where u(f,ξ) are the wavenumber dispersion curves obtained from the B-scan data using the 2D FFT approach; and F(f,ξ) are the Gaussian filters for A_0_ and S_1_ modes. As a result of filtering, the wavenumber dispersion curves were obtained separately for the A_0_ and S_1_ modes. Next, the frequency spectra of these modes were calculated using the following expressions:

(17) $$U_{A_{0}max}\left(f\right)=\underset{\xi }{max}\left(U_{A_{0}}\left(f,\xi \right)\right),$$

(18) $$U_{A_{0}N}\left(f\right)=\frac{U_{A_{0}max}\left(f\right)}{\underset{f}{max}\left(U_{A_{0}max}\left(f\right)\right)},$$

(19) $$U_{S_{1}max}\left(f\right)=\underset{\xi }{max}\left(U_{S_{1}}\left(f,\xi \right)\right),$$

(20) $$U_{S_{1}N}\left(f\right)=\frac{U_{S_{1}max}\left(f\right)}{\underset{f}{max}\left(U_{S_{1}max}\left(f\right)\right)}.$$

As the goal of this study was to compare the simulated spectrum of the A_0_ and S_1_ modes ($U_{A_{0}N}$(f), $U_{S_{1}N}$(f)) with the analytically predicted excitability functions ($H_{A_{0}}$(f_k_), $H_{S_{1}}$(f_k_)), their estimation will be briefly described in the next section.

#### 4.1.2. Estimation of the Excitability Functions for the Considered Problem

**Figure 8:**
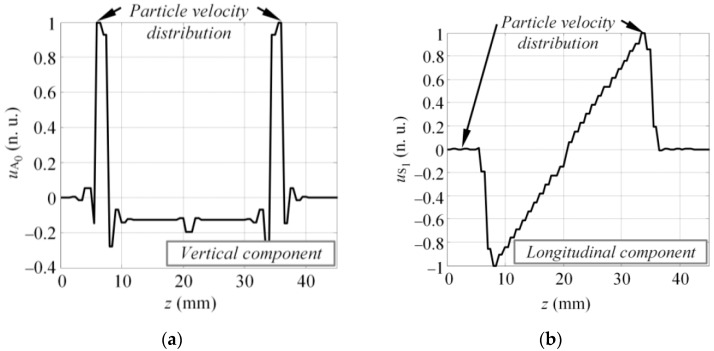
The vertical (**a**) and longitudinal (**b**) component of particle velocity at the excitation surface at the first time instant of the modeling.

To estimate the excitability functions using the technique described in Section 3, the spatial distribution of the particle velocity for the A_0_ and S_1_ modes had to first be defined. In this case, it was presumed that the particle velocity of the vertical component (y) on the active area of the transducer at the first instant of the modeling represented the excitation of the A_0_ mode (see Figure 8a). Similarly, the particle velocity of longitudinal component (z) at the first instant was used to describe the generation of the S_1_ mode (see Figure 8b).

**Figure 9:**
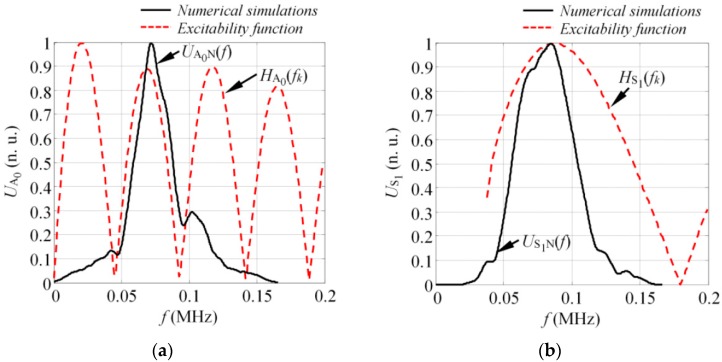
The numerically estimated frequency spectra of the pure A_0_ (**a**) and S_1_ (**b**) mode on a 4 mm GFRP sample (solid line) along with analytically predicted excitability functions (dashed line).

The spatial particle velocity distributions presented in Figure 8 were used to calculate the excitability functions. The same material properties were also used to define the DC for a 4 mm GFRP plate. The excitability functions in this case were estimated in a frequency band up to 200 kHz. Once the calculations were completed, the excitability functions $H_{A_{0}}$(f_k_) and $H_{S_{1}}$(f_k_) were compared to the magnitude spectra of the appropriate mode $U_{A_{0}N}$(f) and $U_{S_{1}N}$(f), estimated by Equations (12) and (13). The comparison of the analytical and numerical results can be seen in Figure 9 where they show good agreement between the numerical calculations and analytical estimation. From the results, it could be concluded that the magnitude spectrum of each guided wave mode was actually the product of the excitation pulse spectrum U_ref_(f) and the excitability function H, which itself depends on the properties of the material, type of excitation, and size of the source. If at least one of the above-mentioned parameters changes, the spectrum of the mode present in the structure will also vary.

### 4.2. Experimental Verification of the Method on the Anisotropic GFRP Plate

To further investigate the feasibility of the proposed excitability function estimation technique, experiments were conducted on GFRP samples possessing the same geometry, mechanical properties and sensor allocation, as described in Section 4.1 and Section 4.1.1. The goal of this study was to compare the experimentally obtained frequency response of the asymmetrical A_0_ mode with the analytically predicted excitability function. 

**Figure 10:**
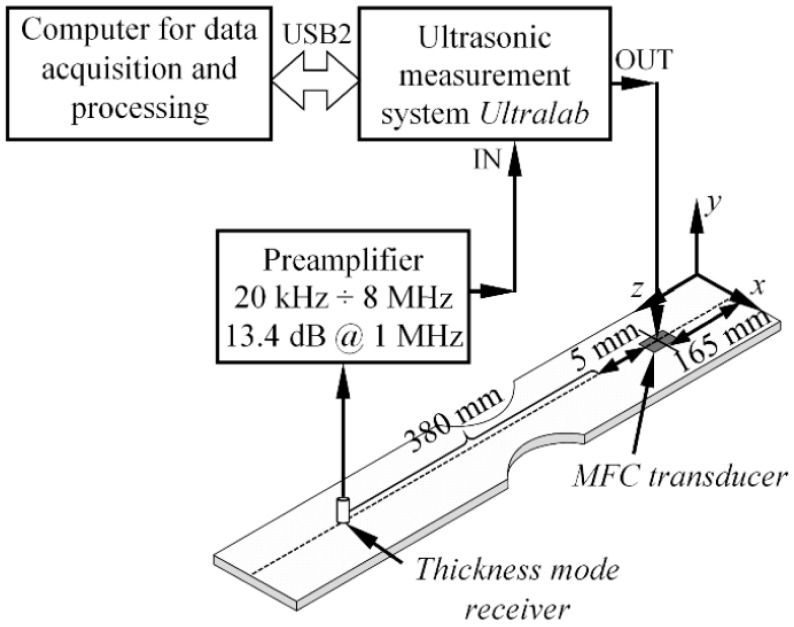
The experimental set-up investigating source influence on guided wave generation.

A MFC actuator (M-2814 P1, Smart Material Corp. Dresden, Germany) was mounted on top of the GFRP sample using a thin layer of wax for coupling as shown in Figure 10. The actuator was driven with a 1 cycle, 50 V square pulse with a central frequency of 80 kHz to generate multiple modes in a wide frequency bandwidth. To collect the experimental data, a custom made low frequency thickness mode transducer (central frequency 240 kHz; bandwidth of the transducer at −6 dB level, 340 kHz) was attached perpendicularly to the surface of the sample and scanned along the wave path of guided waves. The receiver moved away from the transmitter up to 380 mm with a step increment of 1 mm. The initial spacing between the actuator and sensor was equal to 5 mm. To obtain reliable acoustic contact between the sensor and the specimen, a glass textolite protector with a contact area of 3 mm^2^ was used. The waveforms were recorded using a 25 MHz sampling frequency. The response signals were measured eight times and averaged to ensure better a signal to noise ratio. In this way, the B-scan dataset of the out of-plane component was collected at the centerline of the sample. The experimental set-up is graphically illustrated in Figure 10.

**Figure 11:**
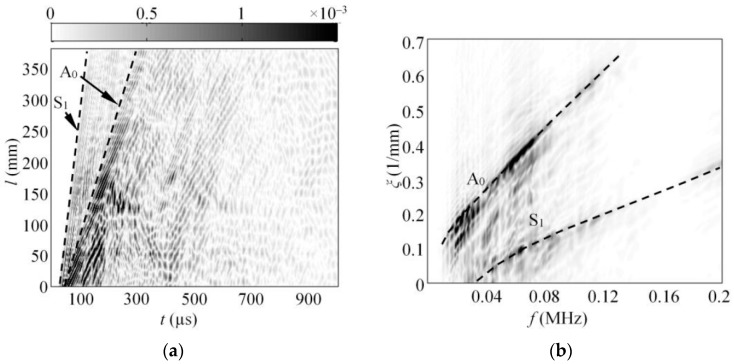
The experimental B-scan of the out-of-plane component of GW along the 4 mm thick GFRP sample (**a**), and the frequency-wavenumber representation of the B-scan data (**b**).

The experimentally obtained raw B-scan u(t,x) image of the out-of-plane component of guided waves is presented in Figure 11a. Analogically, the frequency wavenumber representation of the B-scan data u(f,ξ) showing the modes available in the structure can be seen in Figure 11b. The results in Figure 11a,b shows that two modes were mainly present in the structure, and that the A_0_ was dominant.

**Figure 12:**
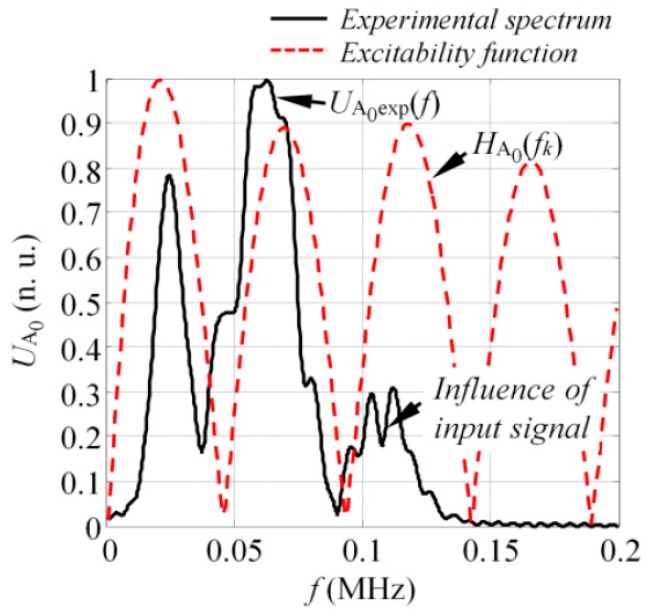
The comparison between the experimental spectrum of the A_0_ mode and the analytical excitability function.

To fulfil the scope of the experiment, the spectrum of the A_0_ mode was extracted from the frequency-wavenumber u(f,ξ) data (Figure 11b) by implementing the procedure described in Section 4.1.1. To calculate the excitability function, it was presumed that the loading distribution required to introduce the A_0_ mode was the same as that presented in Figure 8a. Next, the excitability function of the A_0_ mode was calculated as per Equation (12) in a frequency band up to 200 kHz, assuming that the material properties of the investigated GFRP sample were the same as listed in Section 3. The comparison between the experimentally obtained spectrum of the A_0_ mode $U_{A_{0}\exp}$(f) and the analytical excitability function $H_{A_{0}}$(f_k_) can be seen in Figure 12.

The results showed good agreement between the analytical predictions and experiments. Note that in this case, the transducer was driven by a square pulse of 1 cycle, which means that there might be additional zero harmonics in the frequency spectrum of the A_0_ mode due to the spectrum of input signal. This can be seen in Figure 12 where a local minimum was present at the frequency slightly above 100 kHz.

## 5. Demonstration of the Method Using Linear Phased Array with Variable Aperture

**Figure 13:**
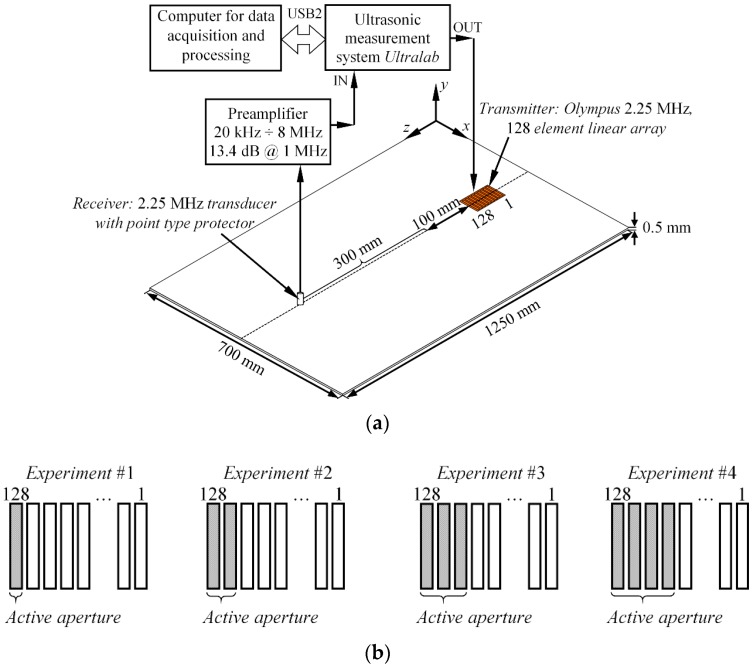
The experimental set-up for investigation of source size influence on guided wave mode spectrum (**a**) and the array aperture configurations used throughout experiments (**b**).

In previous sections, the excitability function estimation technique was introduced and verified. Furthermore, it was demonstrated with numerical simulations and validated with experiments that the excitation type influences the spectrum of each GW mode. In this section, the influence of source size on the forced guided wave excitation was demonstrated and validated. For this purpose, the experiments were carried out on 0.5 mm thick aluminum plate with dimensions of 1250 mm × 700 mm. A material with well-known properties was deliberately selected for this study to simplify the analysis of GW signals. It was estimated that for this type of material, only fundamental modes (A_0_ and S_0_) existed in the frequency band up to 3 MHz. To fulfil the scope of the study, a 2.25 MHz, 128 elements phased array (2.25L128 96 x12 I3 P 2.5HY, Olympus NDT, PA, USA) was used as an actuator and attached to the surface of the plate using oil for acoustic coupling. The array was positioned along the centerline of the sample at a distance of 425 mm to the closest edge of the plate. In total, four independent experiments were carried out (referred as Experiment #1, Experiment #2, etc.) to obtain the response from the structure at different actuator sizes. In the first experiment, only a single element of an array was excited. In each of the subsequent experiments, the active aperture of an array was incremented by adding one neighboring array element to an active aperture, which meant that in the second experiment, two array elements were excited at once, and so on. In each of the experiments, the array was driven by a tone burst of 1 cycle and 200 V with a central frequency of 2.25 MHz. To collect the experimental data, a thickness mode transducer with a point type protector was attached perpendicularly to the surface of the plate and scanned along the wave path of the sample. The initial distance between the array and the receiver was equal to 100 mm, while in each case the receiver was scanned away from the transmitter up to 300 mm with a step increment of 0.1 mm. All waveforms were recorded using the 100 MHz sampling frequency and averaged eight times. The experimental set-up and aperture configurations are presented in Figure 13.

**Figure 14:**
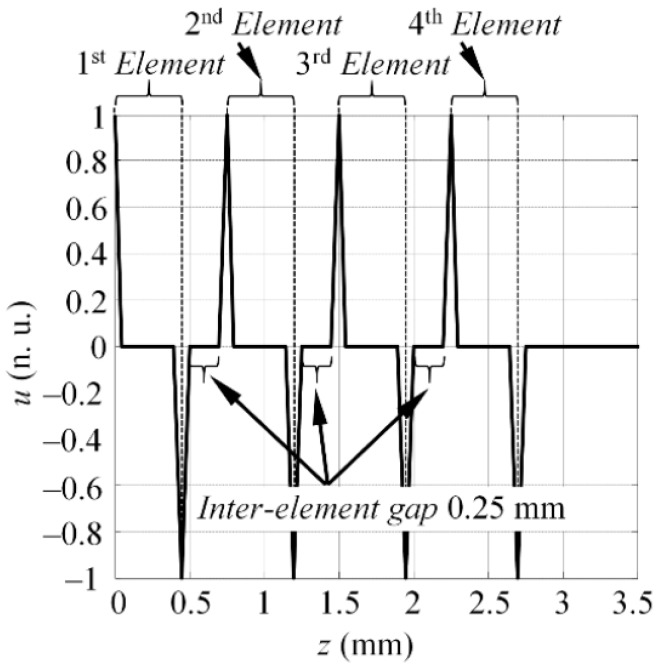
The particle velocity distribution which was required to excite the S_0_ mode in case the four array elements were fired at the same time.

According to the datasheet of the array, the width of each element was equal to 0.5 mm with an inter-element distance of 0.25 mm (pitch 0.75 mm). In this way, the active aperture in Experiment #1 was equal to 0.5 mm, while in each of the subsequent experiments the aperture was equal to—1.25 mm, 2 mm, and 2.75 mm respectively. At the end of the experiments, a total of four B-scan datasets were created, each at a different active aperture of the actuator. In this study, the S_0_ mode was selected as the mode of interest. Thus, the frequency spectrum was estimated from each of the B-scans (namely $U_{S_{0}\exp\#1}$(f), $U_{S_{0}\exp\#2}$(f), $U_{S_{0}\exp\#3}$(f), $U_{S_{0}\exp\#4}$(f)) by employing the procedure described in Section 4.1.1. To estimate the excitability functions, it was presumed that the particle velocity was concentrated at the edges of each element. The example of the spatial particle velocity distribution where four elements were excited at once (Experiment #4) is presented in Figure 14. The particle velocity distributions for the other experiments were defined in a similar fashion, depending on how many elements were fired at the same time. To describe the phase velocities of the guided waves, the following material properties of an aluminum sample were defined: Young’s modulus (72 GPa), and Poisson’s ratio (0.35, the density: 2780 kg/m^3^). In total, four excitability functions (referred as $H_{S_{0}\#1}$(f_k_), $H_{S_{0}\#2}$(f_k_), $H_{S_{0}\#3}$(f_k_), $H_{S_{0}\#4}$(f_k_)) were estimated for each experiment.

**Figure 15:**
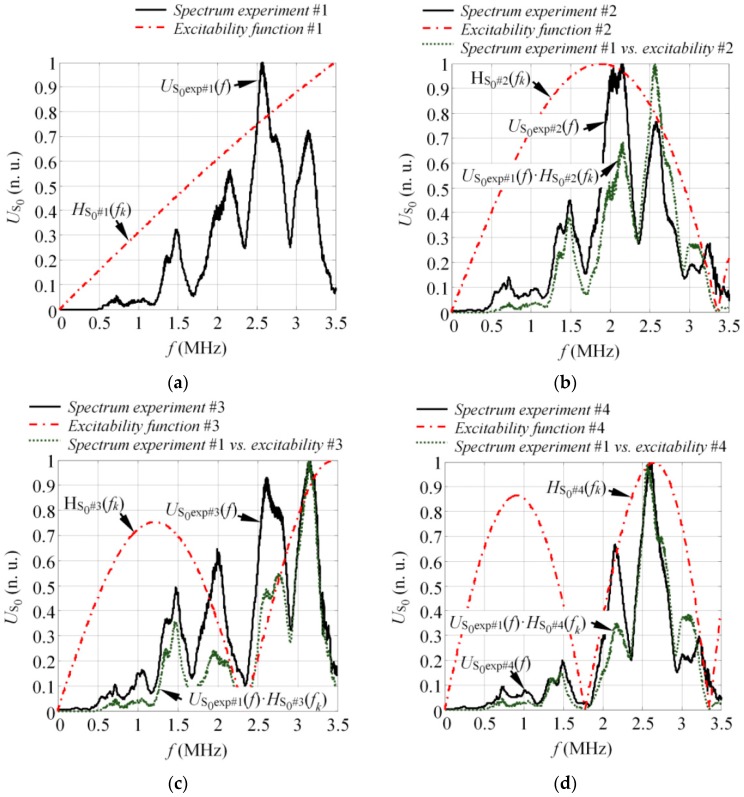
The influence of the source size to the frequency spectrum of the S_0_ mode on the 0.5 mm aluminum sample when the active aperture was 0.5 mm (Experiment #1) (**a**); 1.25 mm (Experiment #2) (**b**); 2 mm (Experiment #3) (**c**); and 2.75 mm (Experiment #4) (**d**).

The frequency spectrum ($U_{S_{0}\exp\#1}$(f)) and the excitability function ($H_{S_{0}\#1}$(f_k_)) of the S_0_ mode when one single element was fired (Experiment #1) can be seen in Figure 15a. The results showed that there were no zero harmonics in the S_0_ mode spectrum, which was caused by the size of the source if only one array element was fired. The results for Experiment #2 can be seen in Figure 15b where in the latter picture, the solid line represents the S_0_ mode spectrum ($U_{S_{0}\exp\#2}$(f)) when two elements were fired simultaneously. The excitability function ($H_{S_{0}\#2}$(f_k_)) was plotted in the dash-dot line, while the round-dot line was the product of $U_{S_{0}\exp\#1}$(f) and $H_{S_{0}\#2}$(f_k_). The results for Experiments #3 and #4 are presented in a similar fashion, where the round-dot line represents $U_{S_{0}\exp\#1}$(f)·$H_{S_{0}\#3}$(f_k_) and $U_{S_{0}\exp\#1}$(f)·$H_{S_{0}\#4}$(f_k_), respectively. The results in Figure 15b–d demonstrate the influence of the source size to the frequency response of the S_0_ mode and observed that the amount of low amplitude frequency components in the excitability function was related to the source width. By knowing the response spectrum when a single array element is fired, each subsequent spectrum at different array apertures can be predicted by calculating the product of $U_{S_{0}\exp\#1}$(f) and the appropriate excitability function $H_{S_{0}\#i}$(f_k_) (i = 2, 3, 4). From the results presented in Figure 15, it was observed that the magnitude of the experimental spectrum $U_{S_{0}\exp\#i}$(f) (solid line) and product of $U_{S_{0}\exp\#1}$(f)·$H_{S_{0}\#i}$(f_k_) (round-dot line) matched at only some frequencies. The calculation of the product $U_{S_{0}\exp\#1}$(f)·$H_{S_{0}\#i}$(f_k_) in all cases was based on the spectrum obtained from Experiment #1 $U_{S_{0}\exp\#i}$(f) and the appropriate excitability function, which depended on array aperture. Thus, in Figure 15b, $U_{S_{0}\exp\#2}$(f) was compared to $U_{S_{0}\exp\#1}$(f)·$H_{S_{0}\#2}$(f_k_). Consequently, in Figure 15c, the $U_{S_{0}\exp\#3}$(f) was compared to $U_{S_{0}\exp\#1}$(f)·$H_{S_{0}\#3}$(f_k_). Hence, the magnitude of energy distribution was different due to a comparison of spectra at different array apertures. Furthermore, in each case, the spectrum was normalized with respect to its own maximum value. As the goal of this comparison was to verify the appearance of low amplitude frequency components at different frequencies due to source size, the differences in magnitude were not further investigated.

## 6. Discussion and Conclusions

In this study, the influence of the source on the frequency response of guided waves was demonstrated and explained. It was shown through numerical simulations and experiments that the frequency response of each guided wave mode was a product of the spectrum of excitation pulse and the excitability function, which itself depended on the type of excitation, material properties, and size of the source. Therefore, the interaction between the guided waves and structural defects depended on the initial properties of each guided wave mode and it became mandatory to predict the response spectrum of each mode to further develop reliable methods for damage detection. In this research, the novel excitability function estimation technique based on Fourier analysis of particle velocity distribution on the excitation area was proposed, which enabled the response amplitude as a function of frequency separately for each GW mode to be estimated. The performance of the proposed technique was demonstrated only for the fundamental asymmetrical and 1st order symmetrical modes; however, the method itself could also be used to predict the excitability functions of other modes. The proposed excitability function estimation technique was validated with numerical simulations and experiments on the GFRP and aluminum samples. The numerical and experimental results showed good agreement with the theoretical predictions. Furthermore, it was demonstrated that the excitability function was related to the wavelength-size ratio of the transducer, therefore the asymmetrical modes (which possess short wavelengths) always had more frequencies with amplitudes close to zero at the same band compared to the symmetrical modes. The technique developed in this study can be further used as a support for existing guided wave signal processing methods to improve their performance, or as one of the predictive modeling tools during the design, implementation, and optimization of structural health monitoring systems.
